# Supplementary figures and images for: The Iron Chelator Desferrioxamine Increases the Efficacy of Bedaquiline in Primary Human Macrophages Infected with BCG
Source: Int J Mol Sci. 2021 Mar 13;22(6):2938. doi: 10.3390/ijms22062938 (PMC8001338; doi:10.3390/ijms22062938)

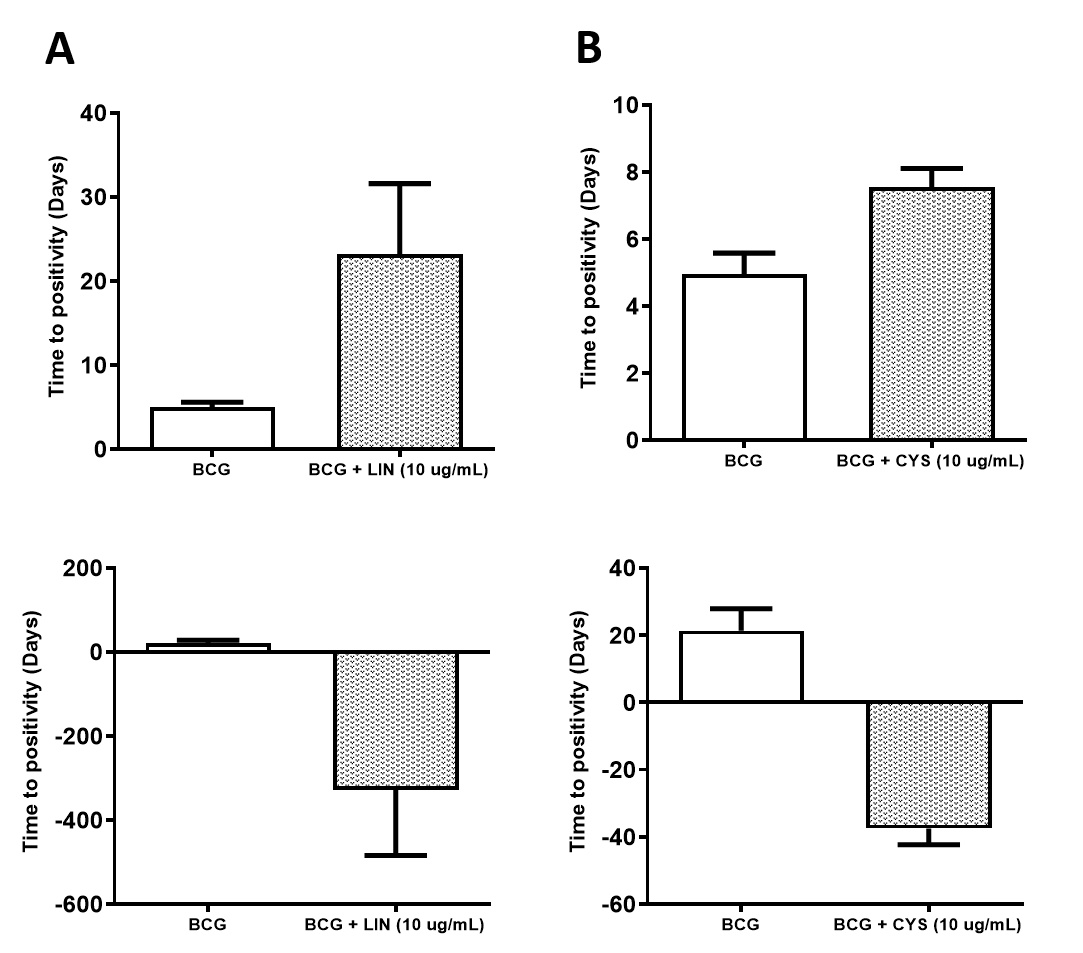

Supplement: Supplementary file 1 [file ijms-22-02938-s001.zip › supplementary materials/Supplementary Figure 1.tif]

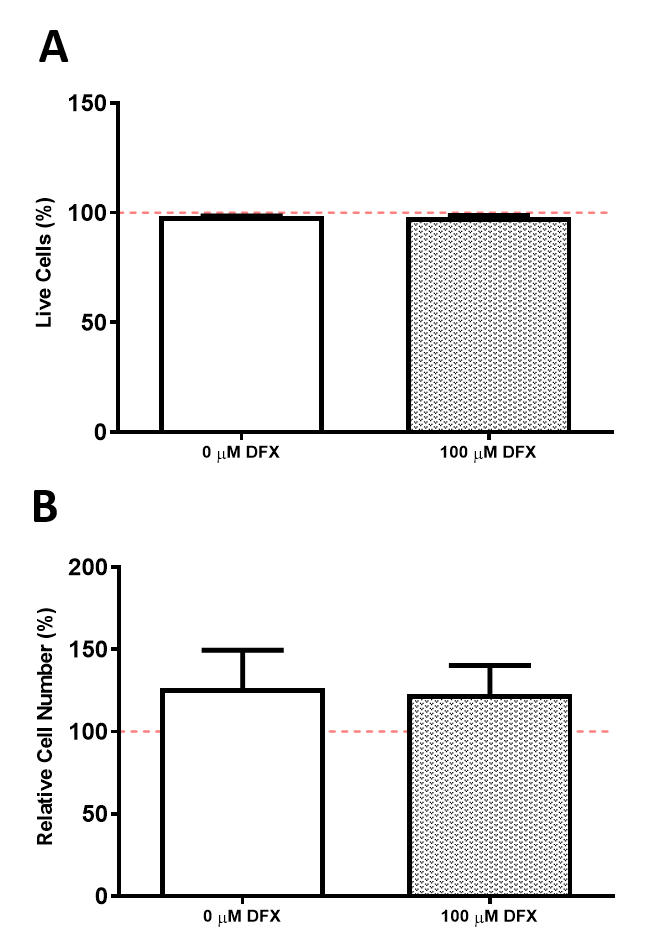

Supplement: Supplementary file 1 [file ijms-22-02938-s001.zip › supplementary materials/Supplementary Figure 2.tif]
